# Namaste Care Family for people with dementia and family−A randomized controlled trial

**DOI:** 10.1002/alz.70495

**Published:** 2025-07-19

**Authors:** Hanneke J. A. Smaling, Karlijn J. Joling, Judith J. M. Rijnhart, Jos W. R. Twisk, Wilco P. Achterberg, Anneke L. Francke, Jenny T. van der Steen

**Affiliations:** ^1^ Department of Public Health and Primary Care Leiden University Medical Center Leiden the Netherlands; ^2^ Department of Public and Occupational Health Amsterdam University Medical Centers, Location VU University Medical Center Amsterdam the Netherlands; ^3^ Leiden University Medical Center University Network for the Care Sector South Holland Leiden the Netherlands; ^4^ Department of Medicine for Older People Amsterdam UMC Amsterdam the Netherlands; ^5^ Amsterdam Public Health, Aging & Later Life Amsterdam the Netherlands; ^6^ College of Public Health University of South Florida Tampa Florida USA; ^7^ Department of Epidemiology and Data Science, Amsterdam Public Health Research Institute VU University Medical Center Amsterdam the Netherlands; ^8^ Netherlands Institute for Health Services Research (NIVEL) Utrecht the Netherlands; ^9^ Radboudumc Alzheimer Center and Department of Primary and Community Care Radboud University Medical Center Nijmegen the Netherlands

**Keywords:** cluster randomized control trial, dementia, family caregivers, Namaste Care, palliative care, quality of life

## Abstract

**INTRODUCTION:**

Few evidence‐based psychosocial interventions target people with advanced dementia.

**METHODS:**

Namaste Care Family is a daily multi‐dimensional care program to improve quality of life (QoL) of people with dementia and their family caregivers. In this cluster‐randomized controlled trial, adjusted linear mixed models were used to analyze effects at 1, 3, 6, and 12 months follow‐up. Ten nursing homes implemented the program (*n* = 116) while nine nursing homes provided care as usual (*n* = 115).

**RESULTS:**

The intervention group showed less discomfort (−1.06; 95% confidence interval [CI] = −1.88 to −0.23) and fewer sentinel events (‐0.40; 95% CI = −0.64 to −0.16), specifically pneumonia (−0.08; 95% CI = −0.14, −0.02). There were no differences in residents’ QoL, challenging behavior, and medication use. There was no effect on positive caregiving experiences, caregiver burden or guilt, whereas conflict with staff was lower at 12 months (−2.04; 95% CI = −4.07 to −0.01).

**DISCUSSION:**

Namaste Care Family has some beneficial effects on residents with dementia and family caregivers.

Clinical Trial Registration: This trial was registered with the CCMO Research with human participants (old ID 5692/new ID 5570).

**Highlights:**

- Namaste Care Family involves family in a daily program for residents with dementia.
- Residents’ quality of life did not improve (primary outcome) due to the program.
- Also, family's positive caregiving experiences did not improve (primary outcome).
- Namaste Care Family reduced discomfort and sentinel events in people with dementia.
- Family caregivers’ conflict with staff over caregiving decreased due to the program.

## 1 BACKGROUND

The provision of care in advanced dementia is generally focused on maximizing comfort.^1^ However, most nursing home residents with dementia experience suboptimal quality of life (QoL).^2^ The symptoms of dementia complicate participation in meaningful activities, engagement in social interactions, and effective communication, potentially resulting in decreased QoL.^3^, ^4^ Family caregivers experience caregiver burden, poor physical health and well‐being, and frustration due to the limited contact with their relative with dementia.^5^, ^6^ Enhancing positive experiences through psychosocial programs may lead to better QoL for nursing home residents and their family caregivers (i.e., family, friends, and volunteers), highlighting the need for evidence‐based psychosocial interventions for people with advanced dementia and their family caregivers.

Namaste Care is a daily care program aimed at improving QoL by connecting with the person with dementia through shared meaningful activities, sensory stimulation, social interaction, and maximizing comfort.^7^ The program addresses the holistic care needs of people with advanced dementia and fits well with expert opinion for what constitutes good palliative care for people with dementia.^8^ Good palliative care for people with dementia comprises multiple domains according to the European Association for Palliative Care, including person‐centered care, psychosocial and spiritual support, family involvement, and education of the healthcare team.^8^ Additionally, professional caregivers perceive the program as a potential driver for palliative care to improve QoL for people with advanced dementia.^9^

Previous studies have provided evidence that individual components of Namaste Care are effective in improving QoL.^10^, ^11^ A realist review^12^ identified what needs to be in place for Namaste Care to be effective for people with advanced dementia. This includes providing a framework for person‐centered care, equipping staff to cope effectively with challenging behavior, and providing structured social and physical stimulation. The underlying belief is the significance of activities that enable moments of meaningful connection for people with advanced dementia.

So far, research on the effects of Namaste Care has included qualitative, small‐scale studies, and studies without a control group. These studies found that Namaste Care can improve QoL, reduce challenging behavior, and possibly decrease psychotropic medication use for nursing home residents.^13^, ^14^, ^15^, ^16^, ^17^, ^18^, ^19^ Staff and family caregivers are generally positive about the program.^9^, ^19^, ^20^ Namaste Care may provide family caregivers with the tools to use activities to meaningfully connect with their relative with dementia.^21^ However, studies examining the effects on family caregivers while actively involving them in the program are scarce. The costs of implementing Namaste Care are relatively low.^20^, ^22^, ^23^ Although the initial studies are promising, randomized controlled trials (RCTs) are needed to further substantiate the evidence of the effectiveness of Namaste Care.

### 1.1 Objectives

The primary objective of this cluster‐RCT was to examine the effects of the Namaste Care Family program on (1) the QoL of nursing home residents with dementia and (2) family caregivers’ positive caregiving experiences. Secondary objectives were to investigate the effects of the program on residents’ (3) discomfort, challenging behavior, psychotropic medication use, and sentinel events and on (4) family caregiver burden, guilt, and conflict in dementia caregiving. Compared to Namaste Care, Namaste Care Family emphasizes family caregiver involvement, that is, providing the sessions together with staff.

## 2 METHODS

### 2.1 Study design

This cluster‐RCT included follow‐up measurements at 1, 3, 6, and 12 months. The trial protocol was published.^24^ Randomization occurred at the nursing home level because the program was structured around groups of residents rather than individuals, and cluster‐randomization reduced contamination risk. An independent statistician randomized matched clusters, consisting of pairs of nursing homes, to either the intervention or control group (1:1 ratio). Nursing homes were matched based on the manager's perceived influence of the nursing home's religious affiliation on the care provided, implemented psychosocial and family participation programs, rural versus urban region, the number of residents on a ward, and whether the ward was part of a small‐scale living arrangement. Matching was done by H.S. and checked by the research team.

The study was performed in accordance with the ethical standards outlined in the 1964 Declaration of Helsinki and its later amendments. The study protocol was reviewed by the Medical Ethics Review Committee of the VU University Medical Center (protocol number: 2016.399) and declared exempt from the Medical Research Involving Human Subjects Act. Data were collected between December 2016 and December 2018. This study was reported in accordance with the CONSORT guideline.^25^

### 2.2 Setting and participants

The study took place in Dutch nursing homes with long‐stay wards specifically for residents with dementia. Nursing homes were eligible if they were willing to collect data irrespective of receiving the intervention. Nursing homes were recruited by sending the manager an email with information about the study. If interested in participating, the manager was asked to complete a short questionnaire to assess the characteristics of the nursing home to facilitate the matching.

After randomization of the nursing homes, participants were recruited by staff who identified eligible residents for the study based on their clinical judgment. Eligible residents had advanced dementia and had a family caregiver willing and able to complete questionnaires. Other inclusion criteria included being unable to participate in regular group activities, or having moderate dementia with challenging behavior, being expected to benefit from the program, or having a family caregiver who was expected to benefit from it according to staff. The nursing home sent family caregivers of eligible residents an invitation letter to participate in the study. After 2 weeks, a reminder was sent. Written informed consent was given by the primary family caregiver. No financial incentive to participate was provided.

**RESEARCH IN CONTEXT**

1. **Systematic review**: The authors reviewed the literature using traditional sources and found that the evidence for the effects of Namaste Care, a daily care program for persons with dementia, on nursing home residents with dementia and their caregivers are promising. However, randomized controlled trials are needed to solidify the evidence‐base.
2. **Interpretation**: No effect of the program was found on residents’ quality of life and family caregivers’ positive caregiving experiences. The program decreased discomfort and sentinel events (including pneumonia). No effects were found on residents’ medication use and on family caregivers’ burden or guilt. Less conflict in dementia caregiving with staff was reported in the intervention group after 12 months.
3. **Future directions**: Future studies should examine the minimal and optimal dose of the program. Also, studies may explore how ‘in the moment’ care for immediate needs as provided by Namaste Care (Family) could be combined with planning for future care needs.

### 2.3 Namaste Care Family

Namaste Care Family is largely similar to the original Namaste Care.^7^ It is a daily multi‐dimensional care program with sensory, psycho‐social, and spiritual components intended to enhance the QoL of people with dementia. Formal pain assessment, increasing staff awareness and responsiveness to distress, and providing comfort to the participants are important aspects of the program. Ideally, 2‐hour sessions are offered twice a day in a calm home‐like room with soft music, pleasant scent, without any distractions. The sessions start with a personalized greeting of each resident. Residents are then comfortably seated and screened for signs of pain. Tempting foods and drinks are offered regularly. Personal care as a meaningful activity alongside individualized activities is offered to connect with the residents. All activities are undertaken at a calm pace and with a gentle, caring touch. Each session ends with a personalized goodbye. Compared to Namaste Care, this adapted program encourages family caregivers to provide the sessions together with the staff.^7^

Nursing staff received a 2‐hour structured training to learn about the principles, purpose, and benefits of the program for people with dementia, their families and staff, and were given practical examples. The training took place after a baseline assessment. Each nursing home received a toolkit with a manual and other tools (e.g., a pain observation instrument, template for resident profiles, example of a weekly Namaste schedule with possible activities for each session, leaflet to recruit volunteers, and a Namaste introduction video)^26^ to help them implement, evaluate, and sustain the program in their nursing home. After 1 month, the “Namaste coordinator” (i.e., person‐in‐charge of all practical aspects of the program) in each nursing home was contacted by H.S. to evaluate the first month of the program and, if needed, to discuss questions and problems. H.S. also observed at least two Namaste sessions in each nursing home, once within the first 3 months, and once after 6 months. Afterward, these sessions were evaluated with the Namaste coordinator.

### 2.4 Data collection

Table 1 presents the instruments used to measure the primary and secondary outcomes. Questionnaires were completed online or on paper. Observations were performed by research assistants who were unfamiliar with the residents. The research assistants received a 2‐hour training in which they practiced the observations with a set of example videos of filmed residents. For all assessments, the observations were ideally scheduled around the same time of the day and performed by the same observer, avoiding mealtime or observation around burdensome procedures. Follow‐up observations in the intervention group were conducted during the Namaste sessions.

**TABLE 1:** Overview of the instruments and time of assessments.

Domain (*rater*)	Instrument	Time of assessment				
		Baseline	Month 1	Month 3	Month 6	Month 12
**Primary outcomes**						
*Person with dementia*						
Quality of life of the person with dementia (*nursing staff*)	Quality of Life in Late‐Stage Dementia (QUALID; 27,28)	x	x	x	x	x
*Family caregiver*						
Positive caregiving experiences (*family caregiver*)	Gain in Alzheimer care INstrument (GAIN; 29)	x	x	x	x	x
**Secondary outcomes**						
*Person with dementia*						
Discomfort–change (*research assistant*)	Discomfort Scale‐Dementia of Alzheimer Type (DS‐DAT; 30)	x	x	x	x	x
Challenging behavior (*nursing staff*)	Neuropsychiatric Inventory Questionnaire (NPI‐Q; 31)	x	x	x	x	x
Medication use (*physician*)	Psychotropic medication	x			x	x
Intercurrent health problems (*physician*)	Sentinel events: pneumonia, urinary tract infection febrile episode, eating or drinking problem, other major medical illness	x			x	x
*Family caregiver*						
Caregiver burden (*family caregiver*)	Zarit's caregiver burden interview (ZBI; 34,35), 7‐item version, and Self‐Rated Burden scale (SRB; 36)	x		x		x
Guilt and conflict in caregiving (*family caregiver*)	Family Perceptions of Caregiving Role (FPCR; 37), subscales "guilt” and "conflict with staff"	x		x		x
Other data						
Demographic information (*family caregiver*)	Socio‐demographic characteristics	x				
Dementia (*physician*)	Type	x				
Dementia severity (*nursing staff*)^a^	Bedford Alzheimer Nursing Severity‐Scale (BANS‐S; 38)	x		x		x

^a^ Deviation from the protocol; the nursing staff filled in the BANS‐S instead of the physician as they have more frequent contact with the residents. This table is an adapted replication from Smaling HJA, Joling KJ, van de Ven PM, et al. Effects of the Namaste Care Family program on quality of life of nursing home residents with advanced dementia and on family caregiving experiences: study protocol of a cluster‐randomized controlled trial. BMJ open. 2018;8(10):e025411.

Blinding of staff and family caregivers was not possible due to the nature of the intervention. The research leads could not be blinded as they were responsible for study coordination, including assisting the intervention group with the implementation of the program. The condition of the nursing home and the goals of the study were not disclosed to the research assistants responsible for performing the resident observations.

### 2.5 Outcomes

#### 2.5.1 Primary outcomes

Residents’ QoL was measured with the Quality of Life in Late‐Stage Dementia (QUALID).^27^, ^28^ This instrument consists of 11 items rated over the previous 7 days along a five‐point Likert scale. Lower scores indicate better QoL.

The Gain in Alzheimer care INstrument (GAIN)^29^ was used to assess family caregivers’ positive caregiving experiences. The scale comprises 10 items that are scored from 0 (disagree a lot) to 4 (agree a lot). Higher scores indicate higher gains.

#### 2.5.2 Secondary outcomes

The Discomfort Scale‐Dementia of Alzheimer Type (DS‐DAT)^30^ was used to observe discomfort. The scale measures duration, intensity, and frequency of 7 negative and 2 positive behaviors for a total score between 0 and 27. Higher scores indicate more discomfort.

Challenging behavior was measured using the Neuropsychiatric Inventory Questionnaire (NPI‐Q).^31^ Nursing staff first indicated the presence of 12 symptoms over the past month. When present, they rated its severity on a three‐point scale. Higher severity sum‐scores indicate more challenging behavior.

Psychotropic medication use was retrieved from medication lists provided by the care team, and classified with the Anatomical Therapeutical Chemical classification (ACT).^32^ The drugs were grouped into antipsychotics (N05A), anxiolytics (N05B), hypnotics and sedatives (N05C), and antidepressants (N06A). A sum‐score of the number of medications per group was calculated.

The elderly care physician, the responsible certified physician of the nursing home, indicated whether the following intercurrent health problems (sentinel events) occurred over the past 6 months: pneumonia, urinary tract infection (UTI), (other) febrile episode, new eating or drinking problem, and other new major medical illness or event.^33^ Sum‐scores were created for the number of sentinel events, pneumonia, and UTIs.

Family caregiver burden was assessed using the seven‐item version of Zarit's Caregiver Burden Interview (ZBI),^34^, ^35^ and the Self‐Rated Burden scale (SRB).^36^ A total score of ≥13 on the ZBI is considered a clinically significant burden.^34^

To measure specific dimensions of family caregiver distress associated with an institutionalized relative with dementia, the subscales “conflict with staff over caregiving” and “guilt from perceived failure in caregiving” of the Family Perceptions of Caregiving Role were used.^37^ Response options form a seven‐point agreement scale.

#### 2.5.3 Other measures

Socio‐demographics of the resident and the family caregiver were assessed using a questionnaire completed by the family caregiver. The severity of dementia was assessed with the Bedford Alzheimer Nursing Severity‐Scale (BANS‐S)^38^, with scores ≥17 indicating severe dementia.^39^

#### 2.5.4 Changes to trial outcomes

The protocol proposed the Positive Experiences Scale (PES)^40^ to measure positive family caregiving experiences. We dropped the PES based on a pilot study ahead of the trial. The PES showed a ceiling effect.^41^ Also, the GAIN had better internal consistency across the assessments (range 0.89–0.94) compared to the PES^40^ (range 0.70–0.79).

### 2.6 Statistical analyses

#### 2.6.1 Sample size calculation

The published protocol^24^ reports a sample size of 192 participants from 16 nursing homes (8 per group) powered to identify medium‐to‐large effect sizes for the primary outcomes.

#### 2.6.2 Data analysis

Because the residuals were sufficiently normally distributed, linear mixed model analyses with a three‐level structure (i.e., repeated measures were clustered within subjects, and subjects were clustered within nursing homes) were used to assess the effect of Namaste Care Family on each of the outcomes at the four follow‐up assessments.

Intervention‐by‐time interaction terms were added to the model to assess whether the effect of the intervention on the outcomes differed over time. Time was considered a categorical variable, that is, represented by three dummy variables. To adjust for potential regression to the mean, all analyses were adjusted for the outcome at baseline. Models with resident outcomes were further adjusted for age, gender, educational level, and dementia severity. Models with family caregiver outcomes were adjusted for age, gender, educational level, the resident's dementia severity, and the type of relationship with the resident.

Cohen's *d* effect sizes were calculated and interpreted based on research in the social sciences: 0.2 represents a small effect, 0.5 a moderate effect, and 0.8 a large effect.^42^, ^43^ Analyses were conducted on an intention‐to‐treat basis. Baseline characteristics were assessed using IBM SPSS Statistics 25 (IBM Corp., Armonk, NY, USA). Mixed model analyses were performed using StataSE 17 (StataCorp LP, CollegeStation, TX, USA).

## 3 RESULTS

### 3.1 Participants

**FIGURE 1:**
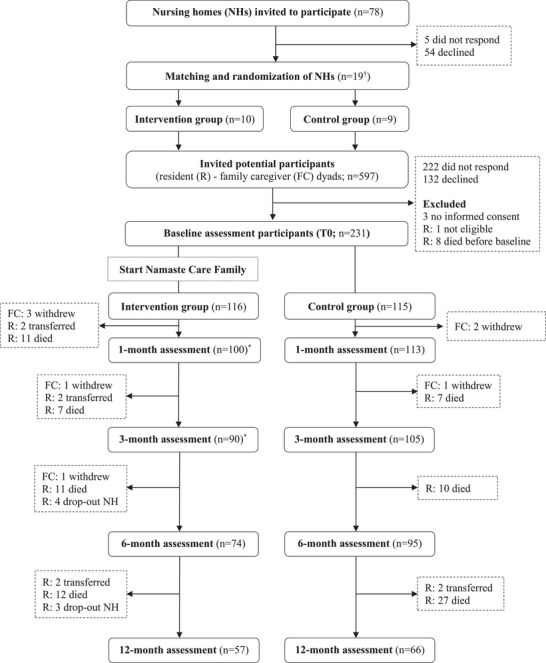
Flow chart nursing home and resident recruitment and participation.

Figure 1 outlines the study flow chart. Of the 78 invited nursing homes; 19 (24%) participated in the study. From these 19 nursing homes, 231 of 597 (39%) eligible residents were included. The intervention group consisted of 116 residents, and 115 residents participated in the control group. Table 2 describes the sample characteristics, including group comparisons. Residents in the intervention group were younger, and they and their family caregivers were more often born in the Netherlands.

**TABLE 2:** Descriptives of the Namaste Care Family group and usual care group at baseline.

Parameter	Namaste Care Family group (intervention)			Usual care group (control)			Group comparison
	N	Mean [SD]/% (*n*)	Min–max	*n*	Mean [SD]/% (*n*)	Min–max	*p*‐value
**Person with dementia**							
Age (years)	110	83.3 [8.1]	56–100	113	86.0 [6.9]	59–100	**0.010** ^*^
Female	116	71% (82)		114	75% (85)		0.510
Born in the Netherlands	108	82% (88)		110	94% (103)		**0.007** ^**^
Educational level							0.374
None or primary school	106	43% (45)		110	35% (39)		
High school preparing for technical/trade school	106	44% (47)		110	54% (59)		
High school preparing for BSc/MSc	106	4% (4)		110	6% (6)		
BSc or MSc degree	106	9% (10)		110	6% (6)		
Dementia severity^a^	115	14.7 [4.8]	7–26	112	15.3 [4.2]	7–26	0.294
Type of dementia							0.543
Alzheimer's disease (AD)	115	50% (57)		111	60% (67)		
Vascular dementia (VD)	115	10% (11)		111	8% (9)		
Mixed type AD–VD	115	19% (22)		111	16% (18)		
Lewy body disease	115	6% (7)		111	5% (5)		
Frontotemporal dementia	115	2% (2)		111	0% (0)		
Alcohol‐related dementia	115	1% (1)		111	0% (0)		
Other	115	13% (15)		111	11% (12)		
Religious background							0.335
Protestant	107	26% (28)		109	24% (26)		
Catholic	107	32% (34)		109	38% (41)		
Other	107	17% (18)		109	7% (8)		
None	107	25% (27)		109	31% (34)		
**Family caregivers**							
Age (years)	108	61.8 [11.1]	34–93	107	63.4 [11.4]	23–94	0.297
Female^b^	116	76% (88)		114	68% (78)		0.240
Born in the Netherlands	108	85% (92)		110	97% (107)		**0.002** ^**^
Educational level							0.112
None or primary school	108	2% (2)		110	8% (9)		
High school preparing for technical/trade school	108	52% (56)		110	52% (57)		
High school preparing for BSc/MSc	108	8% (9)		110	11% (12)		
BSc or MSc degree	108	38% (41)		110	29% (32)		
Religious background							0.219
Protestant	108	15% (16)		110	16% (17)		
Catholic	108	28% (30)		110	36% (39)		
Other	108	19% (19)		110	7% (8)		
None	108	39% (43)		110	42% (46)		
Relation with person with dementia							0.578
Spouse/partner	110	22% (24)		112	17% (19)		
Daughter or son (in law)	110	64% (71)		112	66% (74)		
Other	110	14% (15)		112	17% (19)		

^a^ As measured with the Bedford Alzheimer Nursing Severity‐Scale, ≥ 17 indicating severe dementia.

^b^ Information on gender of four family caregivers in the control and eight in the intervention group became available after publication of the cost‐effectiveness analysis of the Namaste Care Family program by El Alili et al. BMC health services research. 2020.

* *p* < 0.05.

** *p* < 0.01; BSc = Bachelor, MSc = Master.

On average, 6.3 Namaste sessions were provided per week (SD 2.7, range 3–14). Only two nursing homes provided two daily sessions. One nursing home dropped out after 3 months due to problems within the organization while most participants had died. The other nursing home stopped after 6 months due to severe staff shortages and not enough family caregivers to continue the program. On average, family caregivers participated in 3.4 sessions per month. The number of family caregivers who participated in Namaste sessions ranged from 33 (28%) at 1 month after baseline to 15 (13%) at the 12‐month follow‐up.

To investigate whether the four nursing homes that provided at least seven sessions a week experienced higher family involvement compared to the other nursing homes in the intervention group, additional post‐hoc Mann–Whitney *U* tests were performed per assessment. No differences between the two groups were found for the number of times family caregivers participated in the sessions.

Supplement I contains descriptive statistics of the outcomes across the measurements of the total sample, intervention group, and control group. Adjustment for clustering within nursing home units was not needed with intraclass correlation coefficients (ICCs) close to zero (< 0.01).

### 3.2 Effect on the primary outcomes

Table 3 presents the results of the unadjusted and adjusted linear mixed model analyses of the primary resident and family outcome, including the effect sizes for the adjusted models. The overall adjusted mean difference in QoL was 0.69 points (95% confidence interval (CI) = −0.58 to 1.95, *p *= 0.286), indicating no significant overall effect of NCF on QoL. The overall adjusted mean difference in positive caregiving experiences was −0.45 points (95% CI = −1.78 to 0.89, *p *= 0.511), indicating no significant overall effect on positive caregiving experiences. For both primary outcomes, the Cohen's *d* indicated that the effects were very small.

**TABLE 3:** Average difference in the primary outcomes quality of life and positive caregiving experiences across follow‐up assessments at 1, 3, 6, and 12 months.

	Crude (unadjusted) model^a^		Adjusted model^b^		Cohen's *d* adjusted model
Outcome (measure)	*β* (95% CI)	*p*‐value	*β* (95% CI)	*p*‐value	
**Quality of life (QUALID)**					
Overall	0.48 (−0.70, 1.67)	0.432	0.69 (−0.58, 1.95)	0.286	0.09
1 month	0.63 (−0.98, 2.24)	0.443	0.84 (−0.85, 2.53)	0.328	0.11
3 months	1.02 (−0.62, 2.67)	0.222	1.31 (−0.41, 3.04)	0.134	0.17
6 months	0.87 (−0.89, 2.63)	0.333	1.01 (−0.82, 2.84)	0.280	0.13
12 months	−1.55 (−3.54, 0.44)	0.127	−1.52 (−3.60, 0.56)	0.152	−0.18
**Positive caregiving experiences (GAIN)**					
Overall	−0.41 (−1.70, 0.88)	0.533	−0.45 (−1.78, 0.89)	0.511	−0.05
1 month	0.64 (−1.06, 2.34)	0.460	0.50 (−1.24, 2.24)	0.571	0.06
3 months	−0.42 (−2.19, 1.34)	0.639	−0.47 (−2.29, 1.34)	0.610	−0.05
6 months	−1.89 (−3.79, 0.01)	0.052	−1.83 (−3.79, 0.13)	0.067	−0.19
12 months	−0.63 (−2.84, 1.58)	0.577	−0.68 (−2.95, 1.59)	0.558	−0.07

*Note*: The intervention effect is in reference to the control condition. Regression coefficients (*β*) reflect the average difference from baseline during the 12‐month study over the four follow‐up periods (1, 3, 6, and 12 months after intervention).

Abbreviations: CI, confidence interval; GAIN, Gain in Alzheimer Care Instrument; QUALID, Quality of Life in Late‐Stage Dementia (note that higher scores reflect worse quality of life).

^a^ Crude model: adjusted for baseline value of the outcome, and levels time and individual.

^b^ Adjusted model: model with a resident level outcome was further adjusted for age, gender, educational level, and severity of dementia, while the models with a family caregiver level outcome were adjusted for age, gender, and educational level of the family caregiver, severity of dementia of the resident, and the type of relationship between the resident and their family caregiver.

### 3.3 Effect on the secondary resident outcomes

**FIGURE 2:**
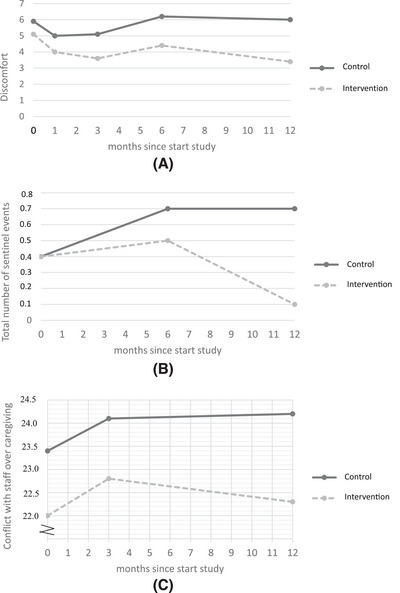
(A) Mean discomfort scores over time for the control and Namaste Care Family (intervention) group. (B) Number of sentinel events over time for the control and Namaste Care Family (intervention) group. (C) Mean conflict with staff over caregiving scores over time for the control and Namaste Care Family (intervention) group.

Table 4 gives the results of the unadjusted and adjusted linear longitudinal multilevel regression analyses for the secondary outcomes, including the effect sizes from the adjusted models. The overall adjusted mean difference of discomfort was −1.06 points (95% CI = −1.88 to −0.23, *p *= 0.012) favoring the intervention condition with Cohen's *d* of −0.22. At 6 months, Cohen's *d* was −0.25, but the mean difference estimate did not reach statistical significance. The overall effect was driven by a difference at 12 months post‐implementation (adjusted mean difference −1.96, 95% CI = −3.55 to −0.37, *p *= 0.016, Cohen's *d* −0.42). Figure 2A visualizes how the mean discomfort scores per group over time differed.

**TABLE 4:** Average differences for secondary outcomes across follow‐up assessments at 1, 3, 6, and 12 months.

	Crude (unadjusted) model^a^		Adjusted model^b^		Cohen's *d* adjusted model
Outcome (measure)	*β* (95% CI)	*p*‐value	*β* (95% CI)	*p*‐value	
**Discomfort (DS‐DAT)**					
Overall	−1.22 (−2.02, −0.42)	**0.003** ^***^	−1.06 (−1.88,−0.23)	**0.012** ^*^	−**0.22**
1 month	−0.65 (−1.85, 0.54)	0.282	−0.67 (−1.89, 0.55)	0.280	−0.14
3 months	−1.05 (−2.30, 0.19)	0.097	−0.69 (−1.96, 0.58)	0.289	−0.15
6 months	−1.46 (−2.80, −0.12)	**0.033** ^*^	−1.31 (−2.67, 0.05)	0.060	−0.25
12 months	−2.18 (−3.73, −0.63)	**0.006** ^**^	−1.96 (‐3.55, −0.37)	**0.016** ^*^	−**0.42**
**Challenging behavior (NPI‐Q)**					
Overall	1.09 (0.08, 2.10)	**0.034** ^*^	0.97 (−0.11, 2.05)	0.079	0.15
1 month	0.47 (−0.88, 1.82)	0.495	0.27 (−1.14, 1.69)	0.705	0.04
3 months	1.36 (−0.01, 2.72)	0.052	1.35 (−0.08, 2.79)	0.065	0.21
6 months	0.91 (−0.55, 2.38)	0.223	0.84 (−0.69, 2.37)	0.282	0.13
12 months	1.91 (0.26, 3.57)	**0.023** ^*^	1.67 (−0.06, 3.40)	0.059	0.26
**Medication use**					
*Antidepressants*					
Overall	0.04 (−0.07, 0.16)	0.442	0.04 (−0.09, 0.16)	0.568	0.07
6 months	0.05 (−0.08, 0.17)	0.481	0.04 (−0.10, 0.17)	0.598	0.07
12 months	0.04 (−0.10, 0.19)	0.533	0.04 (−0.12, 0.19)	0.642	0.07
*Antipsychotics*					
Overall	0.03 (−0.11, 0.17)	0.657	0.02 (−0.13, 0.17)	0.819	0.03
6 months	0.08 (−0.07, 0.24)	0.286	0.06 (−0.10, 0.23)	0.453	0.08
12 months	−0.04 (−0.22, 0.13)	0.637	−0.05 (−0.24, 0.13)	0.590	−0.08
*Anxiolytics*					
Overall	0.02 (−0.09, 0.12)	0.746	0.00 (−0.10, 0.11)	0.943	0
6 months	−0.02 (−0.14, 0.10)	0.771	−0.03 (−0.15, 0.10)	0.686	−0.05
12 months	0.08 (−0.06, 0.21)	0.271	0.05 (−0.09, 0.20)	0.454	0.11
*Hypno‐sedatives*					
Overall	−0.08 (−0.20, 0.04)	0.199	−0.10 (−0.23, 0.03)	0.119	−0.19
6 months	−0.08 (−0.23, 0.06)	0.259	−0.10 (−0.25, 0.05)	0.185	−0.20
12 months	−0.08 (−0.24, 0.09)	0.381	−0.10 (−0.28, 0.08)	0.265	−0.18
*Opioids*					
Overall	0.06 (−0.13, 0.26)	0.521	0.11 (−0.10, 0.32)	0.299	0.15
6 months	0.10 (−0.11, 0.31)	0.369	0.14 (−0.08, 0.36)	0.204	0.19
12 months	0.01 (−0.22, 0.24)	0.927	0.05 (−0.19, 0.29)	0.660	0.07
**Intercurrent health problems**					
*Total sentinel events*					
Overall	−0.37 (−0.59, −0.14)	**0.001** ^***^	−0.40 (−0.64, −0.16)	**0.001** ^***^	−**0.44**
6 months	−0.23 (−0.50, 0.03)	0.088	−0.25 (−0.53, 0.03)	0.080	−0.25
12 months	−0.56 (−0.87, −0.25)	**<0.001** ^****^	−0.61 (−0.94, −0.28)	**<0.001** ^****^	−**0.80**
*Pneumonia*					
Overall	−0.08 (−0.14, −0.03)	**0.002** ^***^	−0.08 (−0.14, ‐0.02)	**0.005** ^**^	−**0.34**
6 months	−0.02 (−0.09, 0.05)	0.615	−0.02 (−0.09, 0.06)	0.648	−0.11
12 months	−0.18 (−0.26, −0.10)	**<0.001** ^****^	−0.18 (−0.27, −0.10)	**<0.001** ^****^	−**0.63**
*Urinary tract infection*					
Overall	−0.02 (−0.06, 0.02)	0.295	−0.03 (−0.07, 0.02)	0.289	−0.16
6 months	−0.03 (−0.08, 0.03)	0.323	−0.03 (−0.09, 0.03)	0.307	−0.15
12 months	−0.01 (−0.08, 0.05)	0.660	−0.02 (−0.09, 0.05)	0.633	−0.13

	Crude (unadjusted) model^a^		Adjusted model^b^		Cohen's *d* adjusted model
	*β* (95% CI)	*p*‐value	*β* (95% CI)	*p*‐value	
**Caregiver burden (ZBI)**					
Overall	−0.43 (−1.27, 0.42)	0.324	−0.58 (−1.44, 0.27)	0.182	−0.12
3 months	−0.37 (−1.31, 0.58)	0.446	−0.49 (−1.45, 0.46)	0.312	−0.10
12 months	−.54 (−1.73, 0.66)	0.379	−0.75 (−1.95, 0.46)	0.224	−0.15
**Self‐rated burden scale (SRB)**					
Overall	−0.18 (−0.67, 0.30)	0.460	−0.29 (−0.79, 0.22)	0.264	−0.12
3 months	−0.16 (−0.71, 0.38)	0.550	−0.27 (−0.83, 0.29)	0.342	−0.11
12 months	−0.21 (−0.90, 0.47)	0.544	−0.31 (−1.01, 0.40)	0.390	−0.13
**Conflict with staff (FPCR subscale)**					
Overall	−1.18 (−2.52, 0.17)	0.086	−0.98 (−2.39, 0.43)	0.175	−0.13
3 months	−0.71 (−2.25, 0.83)	0.364	−0.43 (−2.03, 1.17)	0.596	−0.05
12 months	−2.09 (−4.05, −0.13)	**0.037** ^*^	−2.04 (−4.07, −0.01)	**0.049** ^*^	−**0.28**
**Guilt (FPCR subscale)**					
Overall	−0.13 (−1.00, 0.74)	0.771	−0.14 (−1.04, 0.76)	0.764	−0.03
3 months	−0.10 (−1.08, 0.88)	0.840	−0.06 (−1.07, 0.95)	0.904	−0.01
12 months	−0.16 (−1.41, 1.09)	0.797	−0.27 (−1.54, 1.00)	0.679	−0.06

*Note*: The intervention effect is in reference to the control condition. Regression coefficients (*β*) reflect the average difference from baseline during the 12‐month study over the follow‐up periods (1, 3, 6 and 12 months, 6 and 12 months, or 3 and 12 months after intervention, depending on the secondary outcome).

Abbreviations: CI, confidence interval; DS‐DAT, Discomfort Scale‐Dementia of Alzheimer Type; FPCR, Family Perceptions of Caregiving Role; NPI‐S, Neuropsychiatric Inventory Questionnaire, severity score; SRB, Self‐Rated Burden scale (single 0‐10 scale); UTI, urinary tract infection,; ZBI, Zarit's Caregiver Burden Interview.

^a^ Crude model: adjusted for baseline value of the outcome, and levels time and individual.

^b^ Adjusted model: model with a resident level outcome was further adjusted for adjusted for age, gender, educational level, and severity of dementia, while the models with a family caregiver level outcome was adjusted for age, gender, and educational level of the family caregiver, severity of dementia of the resident, and the type of relationship between the resident and their family caregiver.

* *p *< 0.05 (in bold).

** *p* < 0.01,.

*** *p* < 0.005,

**** *p* < 0.001.

For challenging behavior, the overall adjusted mean difference was 0.97 points (95% CI = −0.11 to 2.05, *p *= 0.079), indicating no overall effect of the intervention on challenging behavior. Cohen's *d* was 0.15. At 3 and 12 months, small effect estimates were observed favoring the control condition (Cohen's *d* 0.21 and 0.26, respectively), although the mean differences were not statistically significant.

The overall adjusted mean difference of the number of antidepressants was 0.04 (95% CI = −0.09 to 0.16, *p *= 0.568), antipsychotics 0.02 (95% CI = −0.13 to 0.17, *p *= 0.819), anxiolytics < 0.00 (95% CI = −0.10 to 0.11, *p *= 0.943), hypno‐sedatives −0.10 (95% CI = −0.23 to 0.03, *p *= 0.119), opioids 0.11 (95% CI = −0.10 to 0.32, *p *= 0.299), indicating no overall significant effect of the intervention on medication use. The Cohen's *d* estimates for the overall effects indicated very small effects.

The overall adjusted mean difference of the total number of sentinel events was ‐0.40 (95% CI = −0.64 to ‐0.16, *p *= 0.001), favoring the intervention condition. The Cohen's *d* was −0.44. The effect was most pronounced at 12 months (adjusted mean difference: −0.61 (95% CI = −0.94 to −0.28, *p *< 0.001, Cohen's *d* −0.80). At 12 months, 90% of the residents of the intervention group had no sentinel events versus 51% in the control group. Figure 2B shows the mean number of sentinel events per assessment, per group. Supplement I shows the results in detail.

More specifically, the overall adjusted mean difference in the number of UTIs was −0.03 (95% CI = −0.07 to 0.02, *p *= 0.289, Cohen's *d* −0.16), indicating no significant overall effect. The overall adjusted mean difference in pneumonia was −0.08 (95% CI = −0.14 to −0.02, *p *= 0.005), which was also most pronounced at 12 months (adjusted mean difference: −0.18, 95% CI = −0.27 to −0.10, *p *< 0.001). The overall effect of the program on pneumonia had an effect size of Cohen's *d* −0.34. At 12 months, none of the residents in the intervention group had had pneumonia in the past 6 months versus 83% in the control group.

### 3.4 Effect on the secondary family caregiver outcomes

There was no overall effect of the program on caregiver burden and guilt (Cohen's *d* estimates were respectively −0.13 and −0.03). The overall adjusted mean difference for conflict with staff over caregiving was −0.98 points (95% CI = −2.39 to 0.43, *p *= 0.175; Cohen's *d* −0.13), indicating no significant overall intervention effect. However, a significant intervention effect of −2.04 points was found at 12 months (95% CI = −4.07 to −0.01, *p *= 0.049, Cohen's *d* −0.28), indicating less conflict with staff over caregiving in the intervention group. Figure 2C shows the mean conflict with staff scores per assessment, per group.

## 4 DISCUSSION

This study examined the effects of Namaste Care Family on resident‐related and family caregiver‐related outcomes compared with usual care. No effects were found on residents’ QoL, challenging behavior, and the number of medications used. Less discomfort and sentinel events (pneumonia) were observed in the Namaste group. No effect was found on family caregivers’ positive caregiving experiences, burden, and guilt. Less conflict in dementia caregiving with staff was reported in the Namaste‐group.

The absence of pneumonia among participants in the Namaste‐group in the last 6 months is striking. This suggests that Namaste Care Family plays a significant role in preventing this life‐threatening condition, even though pneumonia prevention is not a primary focus of the program. Pneumonia is a common terminal event in advanced dementia^44^, ^45^ and often uncomfortable due to distressing symptoms and burdensome treatment,^46^ making this finding highly clinically relevant. Improved nutritional status, the attention paid to residents and the mindful approach in the program may contribute to less choking during eating and drinking, resulting in fewer episodes of pneumonia.

High symptom burden is common in residents with dementia and results in distress and behavioral challenges if undetected.^47^ Discomfort in the Namaste group decreased by almost 2 points, while studies examining DS‐DAT scores at the moment of a treatment decision of pneumonia until 10 days afterwards found reductions of 3.5 to 4 points.^46^, ^48^ Future research should examine whether the decrease in discomfort is associated with the decrease in pneumonia episodes among residents in the program. Supplement II considers potential economic implications of the program in settings where residents are being hospitalized with pneumonia unlike in the Netherlands.

Small non‐significant effects were found on QoL, challenging behavior, and medication use. Although others reported a positive effect of Namaste Care on these outcomes, those studies lacked a control group, used small samples, described trends, and had shorter follow‐up periods, making them difficult to compare.^14^, ^18^, ^49^, ^50^, ^51^, ^52^ For example, Kaasalainen and colleagues reported trends for improved QoL, decreased pain, and reduced medication use. However, only a decrease in the use of antidepressants was statistically significant.^52^ It may be that participating in a study and measuring specific outcomes increases staff's awareness around those outcomes. We observed trends with small effect sizes for challenging behavior at 3 and 12 months in favor of the control group. It could be that challenging behavior is considered more disruptive or more noticeable when present during Namaste sessions, or staff generally paid more attention to residents in the program.

In our qualitative study, family caregivers and staff perceived a positive impact of Namaste Care Family on residents’ well‐being, challenging behavior, mood, engagement, and interaction.^53^ Similar results were reported by others.^54^, ^55^ Possible explanations for the discrepancies between qualitative and quantitative studies are that the effects of the program are more apparent during the Namaste sessions. Interviews allow for asking directly about perceived effects on residents and provide examples of the perceived impact, while the questionnaires are either general (i.e., not specific for Namaste sessions) or ask the participant to complete items while bearing in mind a longer recall period. For medication use, it may be that the physicians who were not much involved in the study exhibited limited responsiveness in reducing medication or that doses rather than the number of medications were revised.

Most studies on Namaste Care used a single‐dimension QoL measure, often the QUALID. Using a single‐score for quality of life or observed well‐being facilitates comparisons between studies, but views on how to measure QoL in dementia are diverse.^56^ QoL is a multifaceted construct, so instruments that include multiple domains may provide a broader understanding of immediate versus more long‐standing impact of the program on residents’ QoL and well‐being.^57^ Future studies should also consider supplementing QoL questionnaires with observational assessments that incorporate multiple aspects of QoL, such as discomfort and positive and negative aspects of the psychosocial environment.^58^

The delivery of two daily 2‐hour sessions was not followed in most nursing homes. This may explain fewer significant outcomes than anticipated. Two‐hour sessions may not be feasible for all residents.^53^ To ensure Namaste Care is practical and achieves the intended outcomes, it is crucial to comprehensively document its components, processes, and outcomes to facilitate evidence‐based modifications to the implementation of Namaste Care. Nevertheless, the daily Namaste sessions still ensure more time for meaningful activities for residents than in other psychosocial interventions. There is a need to investigate the minimal dose of the program for it to have beneficial effects, and to assess optimal doses. Future studies should examine the relationship between adherence to the program and observed outcomes. A next step would be to examine subgroups in which the program is more or less effective, to identify residents who may benefit most from participating.

The challenge of adhering to the session planning is not unique to our study. Others identified similar challenges in implementing Namaste Care.^59^ Their explanations were in line with those mentioned in our study, such as time constraints and limited staffing.^59^, ^60^ A more flexible approach to implementation might increase implementation success, once the optimal dose has been established. For example, by adopting flexible scheduling to accommodate the specific constraints of each nursing home and to involve family and volunteers more systematically, while maintaining the core components of Namaste Care.^59^ Physical and social opportunities and psychological capability are common targets for change to overcome barriers and leverage facilitators.^61^ Besides structured staff education^62^ and training, we recommend periodic monitoring of activities every 4 to 8 weeks to ensure intervention fidelity. A train‐the‐trainer concept should be considered to ensure in‐house knowledge, continuous involvement, adherence, and education of new staff, volunteers, and family.

We identified significant effects 12 months after the start of Namaste Care Family, while most studies examining effects of psychosocial interventions had already stopped measuring. The implementation of a multicomponent program may need time for the full effect to become apparent. Raising awareness and culture change also need time. Our findings highlight the relevance of extended measurement periods.

It would be important to examine if and how active family participation in the Namaste sessions and family caregiver outcomes are related. Involved families facilitate the delivery of the program.^55^, ^59^ However, family involvement was relatively low.^23^ This may explain why no effects on caregiver burden, positive caregiving experiences, and guilt from perceived failure in caregiving were found. It may be worth considering organizing Namaste sessions in the evening to facilitate family involvement for relatives and volunteers with day jobs and school‐going grandchildren. Other recommendations include invitations to specific activities, regular updates on the program, discussions about family involvement with family caregivers when residents are admitted to the nursing home, and provide tools like the Family Carer Decision Support intervention (FCDS; “mySupport”)^63^ to facilitate communication and participation from families. FCDS is combined with Namaste Care in the In‐Touch study.^64^ Family involvement should be encouraged, although the extent must be tailored to and determined together with the family caregiver, taking into account their wishes, abilities, needs, caregiver burden, and their relationship with their relative.^54^, ^65^ Tasseron et al.^65^ presented an overview of the facilitators for and barriers to family involvement in Namaste Care Family.

A possible explanation for the relatively low family involvement is that the program reassured family in that they knew their relative received meaningful activities and personal attention, which allowed family some respite time which can be positive.^52^, ^54^ While most family caregivers enjoyed participating in the program, for some, participation was difficult.^53^ This may be reason for them not to partake in the sessions. Alternatively, family may feel guilty for having their relative admitted to the nursing home. Guilt and shame may cause family to distance themselves from their relative with dementia.^66^ More research into factors affecting sustainable family involvement is needed. Further research should examine mechanisms that explain the effect of the program on reducing conflict between family and staff. Effects might be explained by better communication between family and staff due to the program. Qualitative research should further explore how Namaste Care Family affects caregiver–staff dynamics beyond quantitative measures.

Strengths include the cluster‐RCT design and sufficiently powered sample to detect the expected medium to large effect sizes. This is the first RCT examining the effects of Namaste Care Family on a broad range of resident and family caregiver outcomes using multiple reliable and valid instruments. Limitations are, the low family involvement and that only two nursing homes adhered to the planned session frequency. Surrogate measures for resident outcomes were unavoidable within this population. Due to the nature of the intervention, it was impossible to blind staff and families. We asked a long‐term commitment from the nursing homes, potentially leading to selection bias. We cannot rule out control group nursing homes having engaged in other ways to improve their residents’ QoL. Expanding the study across different cultural contexts would strengthen generalizability.

To conclude, Namaste Care Family improves the well‐being of residents with dementia by reducing sentinel events, pneumonia, and discomfort. The program resulted in less conflict between family caregivers and staff. Namaste Care Family facilitates meaningful connections between family caregivers and residents, and encourages care partnerships between staff and families.^20^, ^53^, ^54^, ^67^

## AUTHOR CONTRIBUTIONS

Jenny T. van der Steen obtained funding for the research. All authors have contributed to the design of the study. Hanneke J. A. Smaling was responsible for data collection and performed the descriptive analyses. Judith J. M. Rijnhart and Hanneke J. A. Smaling wrote the statistical analyses section. Jos W. R. Twisk conducted the analyses for the primary and secondary outcomes. The first draft of this manuscript was produced by Hanneke J. A. Smaling and all authors have reviewed, edited, and approved the final version. Jenny T. van der Steen and Hanneke J. A. Smaling are the guarantors. The corresponding author attests that all listed authors meet authorship criteria and that no others meeting the criteria have been omitted.

## CONFLICT OF INTEREST STATEMENT

H.S. became an unpaid member and associate director of Namaste Care International after this study was completed. The other authors have nothing to disclose. Author disclosures are available in the Supplementary Information.

## CONSENT STATEMENT

Written informed consent was given by the primary family caregiver of the nursing home resident with dementia.

## Supporting information



Supporting Information

Supporting Information

Supporting Information
