# Supplementary material for: Namaste Care Family for people with dementia and family−A randomized controlled trial
Source: Alzheimers Dement. 2025 Jul 19;21(7):e70495. doi: 10.1002/alz.70495 (PMC12276077; doi:10.1002/alz.70495)
Supplement: Supplementary file 1 — Supporting Information [file ALZ-21-e70495-s001.docx]

**Supplementary materials**

Smaling HJA, Joling KJ, Rijnhart JJM, Twisk JWR, Achterberg WP, Francke AL, van der Steen JT. Namaste Care Family for people with dementia and

family - a randomized controlled trial.

**Supplement I:** Descriptives of the primary and secondary outcomes over time of nursing home residents with dementia and their family caregivers in the Dutch Namaste study.

| **Primary outcomes** | | **Total sample** | | | **Namaste group** | | | | **Control group** | | | | **Group comparisons^†^** |
| --- | --- | --- | --- | --- | --- | --- | --- | --- | --- | --- | --- | --- | --- |
|  |  | **N** | **Mean (SD)** | **range** | **n** | **Mean (SD)** | | **range** | **n** | **Mean (SD)** | **range** | |  |
| QUALID | T0 | 228 | 23.3 (8.1) | 11-52 | 115 | 24.2 (8.5) | | 12-48 | 113 | 22.3 (7.7) | 11-52 | | *z* = -1.50 |
| *Quality of life* | T1 | 201 | 23.8 (7.7) | 11-47 | 101 | 24.3 (7.7) | | 12-47 | 100 | 23.2 (7.7) | 11-44 | | *z* = -1.02 |
|  | T2 | 193 | 23.1 (7.7) | 11-43 | 92 | 24.0 (7.7) | | 13-42 | 101 | 22.3 (7.7) | 11-43 | | *z* = -1.51 |
|  | T3 | 166 | 22.1 (7.6) | 11-45 | 73 | 23.1 (8.1) | | 11-45 | 93 | 21.4 (7.2) | 11-41 | | *z* = -1.29 |
|  | T4 | 123 | 23.0 (8.4) | 11-45 | 57 | 22.7 (8.1) | | 11-42 | 66 | 23.2 (8.8) | 11-45 | | *z* = -0.18 |
| GAIN | T0 | 215 | 23.9 (8.6) | 0-40 | 107 | 24.9 (8.6) | | 0-40 | 108 | 22.9 (8.6) | 0-40 | | t(213)= -1.6 |
| *Positive caregiving experiences* | T1 | 190 | 24.1 (8.7) | 0-40 | 90 | 25.2 (8.2) | | 4-40 | 100 | 23.0 (9.1) | 0-40 | | t(188)= -1.7 |
|  | T2 | 176 | 23.3 (8.8) | 0-40 | 79 | 23.8 (8.9) | | 0-40 | 97 | 22.9 (8.7) | 0-40 | | t(174)= -0.7 |
|  | T3 | 148 | 23.6 (9.5) | 0-40 | 64 | 23.0 (10.0) | | 0-40 | 84 | 24.0 (9.1) | 0-40 | | t(146)= 0.6 |
|  | T4 | 103 | 23.3 (9.5) | 0-40 | 47 | 23.8 (9.6) | | 0-40 | 56 | 22.8 (9.5) | 0-40 | | t(101)= -0.5 |
| **Secondary outcomes  *Person with dementia*** |  | **N** | **Mean (SD)** | **range** | **n** | **Mean (SD)** | | **range** | **n** | **Mean (SD)** | **range** | | **Group comparisons^†^** |
| DS-DAT | T0 | 229 | 5.5 (5.0) | 0-23 | 115 | 5.1 (4.8) | | 0-22 | 114 | 5.9 (5.1) | 0-23 | | *z* = -1.26 |
| *Discomfort* | T1 | 211 | 4.6 (4.6) | 0-27 | 99 | 4.0 (4.3) | | 0-22 | 112 | 5.0 (4.9) | 0-27 | | *z* = -1.63 |
|  | T2 | 193 | 4.4 (4.5) | 0-21 | 90 | 3.6 (4.5) | | 0-21 | 103 | 5.1 (4.4) | 0-19 | | ***z* = -3.18****** |
|  | T3 | 167 | 5.4 (5.1) | 0-22 | 74 | 4.4 (5.1) | | 0-19 | 93 | 6.2 (5.1) | 0-22 | | ***z* = -3.01***** |
|  | T4 | 121 | 4.7 (4.7) | 0-19 | 57 | 3.4 (3.8) | | 0-14 | 64 | 6.0 (5.1) | 0-19 | | ***z* = -3.09***** |
| NPI-Q | T0 | 227 | 8.0 (6.8) | 0-32 | 115 | 8.3 (6.7) | | 0-31 | 112 | 7.7 (7.0) | 0-32 | | *z* = -0.97 |
| *Behavioural symptoms of dementia* | T1 | 199 | 7.0 (6.7) | 0-31 | 101 | 7.0 (6.3) | | 0-27 | 98 | 6.9 (7.1) | 0-31 | | *z* = -0.65 |
|  | T2 | 193 | 6.6 (6.3) | 0-30 | 92 | 7.4 (6.8) | | 0-30 | 101 | 5.8 (5.8) | 0-29 | | *z* = -1.76 |
|  | T3 | 165 | 6.1 (6.2) | 0-31 | 73 | 7.0 (6.9) | | 0-31 | 93 | 5.4 (5.6) | 0-23 | | *z* = -1.76 |
|  | T4 | 122 | 6.6 (6.4) | 0-29 | 56 | 8.3 (7.1) | | 0-29 | 66 | 5.1 (5.4) | 0-20 | | ***z* = -2.90***** |
|  |  | **Total sample**  **Count** | | **n (%)** | **Namaste group**  **Count** | | | **n (%)** | **Control group**  **Count** | | **n (%)** | | **Group comparisons^†^** |
| (Number of) sentinel events ^‡^ | T0 | 0 |  | 154 (68%) | 0 |  | | 77 (67%) | 0 |  | 77 (69%) | | *z* = -0.21 |
|  |  | 1 |  | 54 (24%) | 1 |  | | 30 (26%) | 1 |  | 24 (22%) | |  |
|  |  | 2 |  | 12 (5%) | 2 |  | | 7 (6%) | 2 |  | 5 (5%) | |  |
|  |  | 3 |  | 6 (3%) | 3 |  | | 1 (1%) | 3 |  | 5 (5%) | |  |
|  | T3 | 0 |  | 107 (64%) | 0 |  | | 50 (67%) | 0 |  | 57 (61%) | | *z* = -1.07 |
|  |  | 1 |  | 39 (23%) | 1 |  | | 19 (25%) | 1 |  | 20 (22%) | |  |
|  |  | 2 |  | 13 (8%) | 2 |  | | 4 (5%) | 2 |  | 9 (10%) | |  |
|  |  | 3 |  | 4 (2%) | 3 |  | | 1 (1%) | 3 |  | 3 (3%) | |  |
|  |  | 4 |  | 3 (2%) | 4 |  | | 1 (1%) | 4 |  | 2 (2%) | |  |
|  |  | 5 |  | 2 (1%) | 5 |  | | - | 5 |  | 2 (2%) | |  |
|  | T4 | 0 |  | 84 (69%) | 0 |  | | 51 (90%) | 0 |  | 33 (51%) | | ***z* = -4.65****** |
|  |  | 1 |  | 25 (21%) | 1 |  | | 5 (9%) | 1 |  | 20 (31%) | |  |
|  |  | 2 |  | 9 (7%) | 2 |  | | 1 (1%) | 2 |  | 8 (12%) | |  |
|  |  | 3 |  | 4 (3%) | 3 |  | | - | 3 |  | 4 (6%) | |  |
| (Number of) pneumonia | T0 | 0 |  | 215 (95%) | 0 |  | | 110 (96%) | 0 |  | 105 (95%) | | P = 0.765 |
|  |  | 1 |  | 11 (5%) | 1 |  | | 5 (4%) | 1 |  | 6 (5%) | |  |
|  | T3 | 0 |  | 162 (96%) | 0 |  | | 73 (97%) | 0 |  | 89 (96%) | | P = 0.693 |
|  |  | 1 |  | 6 (4%) | 1 |  | | 2 (3%) | 1 |  | 4 (4%) | |  |
|  | T4 | 0 |  | 111 (91%) | 0 |  | | 57 (100%) | 0 |  | 54 (83%) | | **P < 0.001****** |
|  |  | 1 |  | 11 (9%) | 1 |  | | - | 1 |  | 11 (17%) | |  |
| (Number of) urinary tract infections | T0 | 0 |  | 219 (97%) | 0 |  | | 114 (99%) | 0 |  | 105 (95%) | | P = 0.094 |
|  |  | 1 |  | 5 (2%) | 1 |  | | 1 (1%) | 1 |  | 4 (4%) | |  |
|  |  | 2 |  | 2 (1%) | 2 |  | | - | 2 |  | 2 (2%) | |  |
|  | T3 | 0 |  | 161 (96%) | 0 |  | | 73 (97%) | 0 |  | 88 (95%) | | P = 0.463 |
|  |  | 1 |  | 7 (4%) | 1 |  | | 2 (3%) | 1 |  | 5 (5%) | |  |
|  | T4 | 0 |  | 119 (98%) | 0 |  | | 56 (98%) | 0 |  | 63 (97%) | | P = 1.000 |
|  |  | 1 |  | 3 (2%) | 1 |  | | 1 (2%) | 1 |  | 2 (3%) | |  |
| (Number of prescribed)  anxiolytics | T0 | 0 |  | 181 (80%) | 0 |  | | 93 (81%) | 0 |  | 88 (79%) | | *z* = -0.42 |
|  |  | 1 |  | 40 (18%) | 1 |  | | 19 (17%) | 1 |  | 21 (19%) | |  |
|  |  | 2 |  | 6 (3%) | 2 |  | | 3 (3%) | 2 |  | 3 (3%) | |  |
|  | T3 | 0 |  | 138 (83%) | 0 |  | | 60 (81%) | 0 |  | 78 (84%) | | *z* = -0.38 |
|  |  | 1 |  | 20 (12%) | 1 |  | | 11 (15%) | 1 |  | 9 (10%) | |  |
|  |  | 2 |  | 7 (4%) | 2 |  | | 2 (3%) | 2 |  | 5 (5%) | |  |
|  |  | 3 |  | 1 (1%) | 3 |  | | 1 (1%) | 3 |  | - | |  |
|  |  | 4 |  | 1 (1%) | 4 |  | | - | 4 |  | 1 (1%) | |  |
|  | T4 | 0 |  | 104 (85%) | 0 |  | | 47 (83%) | 0 |  | 57 (86%) | | *z* = 0.55 |
|  |  | 1 |  | 17 (14%) | 1 |  | | 9 (16%) | 1 |  | 8 (12%) | |  |
|  |  | 2 |  | 1 (1%) | 2 |  | | - | 2 |  | 1 (2%) | |  |
|  |  | 3 |  | 1 (1%) | 3 |  | | 1 (2%) | 3 |  | - | |  |
| (Number of prescribed)  anti-depressants | T0 | 0 |  | 163 (72%) | 0 |  | | 84 (73%) | 0 |  | 79 (71%) | | *z* = -0.78 |
|  |  | 1 |  | 50 (22%) | 1 |  | | 29 (25%) | 1 |  | 21 (19%) | |  |
|  |  | 2 |  | 9 (4%) | 2 |  | | - | 2 |  | 9 (8%) | |  |
|  |  | 3 |  | 3 (1%) | 3 |  | | 1 (1%) | 3 |  | 2 (2%) | |  |
|  |  | 4 |  | 1 (<1%) | 4 |  | | - | 4 |  | 1 (1%) | |  |
|  |  | 5 |  | 1 (<1%) | 5 |  | | 1 (1%) | 5 |  | - | |  |
|  | T3 | 0 |  | 120 (72%) | 0 |  | | 53 (72%) | 0 |  | 67 (72%) | | *z* = -0.05 |
|  |  | 1 |  | 40 (24%) | 1 |  | | 18 (24%) | 1 |  | 22 (24%) | |  |
|  |  | 2 |  | 7 (4%) | 2 |  | | 3 (4%) | 2 |  | 4 (4%) | |  |
|  | T4 | 0 |  | 87 (71%) | 0 |  | | 40 (70%) | 0 |  | 47 (71%) | | *z* = -0.02 |
|  |  | 1 |  | 31 (25%) | 1 |  | | 16 (28%) | 1 |  | 15 (23%) | |  |
|  |  | 2 |  | 4 (3%) | 2 |  | | - | 2 |  | 4 (6%) | |  |
|  |  | 3 |  | 1 (1%) | 3 |  | | 1 (2%) | 3 |  | - | |  |
| (Number of prescribed)  anti-psychotics | T0 | 0 |  | 167 (74%) | 0 |  | | 81 (70%) | 0 |  | 86 (77%) | | *z* = -0.93 |
|  |  | 1 |  | 46 (20%) | 1 |  | | 28 (24%) | 1 |  | 18 (16%) | |  |
|  |  | 2 |  | 8 (4%) | 2 |  | | 3 (3%) | 2 |  | 5 (5%) | |  |
|  |  | 3 |  | 3 (1%) | 3 |  | | 1 (1%) | 3 |  | 2 (1%) | |  |
|  |  | 4 |  | 2 (1%) | 4 |  | | 1 (1%) | 4 |  | 1 (1%) | |  |
|  |  | 5 |  | 1 (<1%) | 5 |  | | 1 (1%) | 5 |  | - | |  |
|  | T3 | 0 |  | 119 (71%) | 0 |  | | 48 (65%) | 0 |  | 71 (76%) | | *z* = -1.53 |
|  |  | 1 |  | 35 (21%) | 1 |  | | 20 (27%) | 1 |  | 15 (16%) | |  |
|  |  | 2 |  | 9 (5%) | 2 |  | | 3 (4%) | 2 |  | 6 (7%) | |  |
|  |  | 3 |  | 3 (2%) | 3 |  | | 3 (4%) | 3 |  | - | |  |
|  |  | 4 |  | 1 (1%) | 4 |  | | - | 4 |  | 1 (1%) | |  |
|  | T4 | 0 |  | 93 (76%) | 0 |  | | 42 (74%) | 0 |  | 51 (77%) | | *z* = -0.30 |
|  |  | 1 |  | 25 (20%) | 1 |  | | 14 (25%) | 1 |  | 11 (17%) | |  |
|  |  | 2 |  | 4 (3%) | 2 |  | | 1 (1%) | 2 |  | 3 (5%) | |  |
|  |  | 3 |  | - | 3 |  | | - | 3 |  | - | |  |
|  |  | 4 |  | 1 (1%) | 4 |  | | - | 4 |  | 1 (2%) | |  |
| (Number of prescribed)  hypno-sedatives | T0 | 0 |  | 191 (84%) | 0 |  | | 103 (90%) | 0 |  | 88 (79%) | | ***z* = -2.30*** |
|  |  | 1 |  | 31 (14%) | 1 |  | | 11 (10%) | 1 |  | 20 (18%) | |  |
|  |  | 2 |  | 4 (2%) | 2 |  | | 1 (1%) | 2 |  | 3 (3%) | |  |
|  |  | 3 |  | 1 (<1%) | 3 |  | | - | 3 |  | 1 (1%) | |  |
|  | T3 | 0 |  | 137 (82%) | 0 |  | | 65 (88%) | 0 |  | 72 (77%) | | *z* = -1.82 |
|  |  | 1 |  | 26 (16%) | 1 |  | | 9 (12%) | 1 |  | 17 (18%) | |  |
|  |  | 2 |  | 3 (2%) | 2 |  | | - | 2 |  | 3 (3%) | |  |
|  |  | 3 |  | 1 (1%) | 3 |  | | - | 3 |  | 1 (1%) | |  |
|  | T4 | 0 |  | 103 (84%) | 0 |  | | 51 (90%) | 0 |  | 52 (79%) | | *z* = -1.61 |
|  |  | 1 |  | 16 (13%) | 1 |  | | 5 (9%) | 1 |  | 11 (17%) | |  |
|  |  | 2 |  | 3 (2%) | 2 |  | | 1 (2%) | 2 |  | 2 (3%) | |  |
|  |  | 3 |  | - | 3 |  | | - | 3 |  | 1 (2%) | |  |
|  |  | 4 |  | 1 (1%) | 4 |  | | - | 4 |  | (%) | |  |
| (Number of prescribed)  opioids | T0 | 0 |  | 171 (75%) | 0 |  | | 87 (76%) | 0 |  | 84 (75%) | | *z* = -0.19 |
|  |  | 1 |  | 42 (19%) | 1 |  | | 22 (19%) | 1 |  | 20 (18%) | |  |
|  |  | 2 |  | 9 (4%) | 2 |  | | 4 (4%) | 2 |  | 5 (5%) | |  |
|  |  | 3 |  | 5 (2%) | 3 |  | | 2 (2%) | 3 |  | 3 (3%) | |  |
|  | T3 | 0 |  | 119 (71%) | 0 |  | | 53 (72%) | 0 |  | 66 (71%) | | *z* = -0.14 |
|  |  | 1 |  | 26 (16%) | 1 |  | | 9 (12%) | 1 |  | 17 (18%) | |  |
|  |  | 2 |  | 20 (12%) | 2 |  | | 11 (15%) | 2 |  | 9 (10%) | |  |
|  |  | 3 |  | 2 (2%) | 3 |  | | 1 (1%) | 3 |  | 1 (1%) | |  |
|  | T4 | 0 |  | 93 (76%) | 0 |  | | 43 (75%) | 0 |  | 50 (76%) | | *z* = -0.05 |
|  |  | 1 |  | 22 (18%) | 1 |  | | 11 (19%) | 1 |  | 11(17%) | |  |
|  |  | 2 |  | 4 (3%) | 2 |  | | 2 (4%) | 2 |  | 2 (3%) | |  |
|  |  | 3 |  | 4 (3%) | 3 |  | | 1 (2%) | 3 |  | 3 (5%) | |  |
| **Secondary outcomes**  ***Family caregiver*** |  | **N** | **Mean (SD)** | **range** | **n** | | **Mean (SD)** | **range** | **n** | **Mean (SD)** | | **range** | **Group comparisons^†^** |
| ZBI | T0 | 216 | 12.9 (5.7) | 7-34 | 108 | 13.5 (6.2) | | 7-34 | 108 | 12.3 (5.2) | 7-34 | | *z* = -1.01 |
| *Caregiver burden* | T2 | 176 | 12.2 (4.9) | 7-32 | 79 | 12.4 (5.2) | | 7-32 | 97 | 12.0 (4.6) | 7-27 | | *z* = -0.30 |
|  | T4 | 103 | 11.9 (4.9) | 7-32 | 47 | 12.0 (5.1) | | 7-32 | 56 | 11.7 (4.7) | 7-27 | | *z* = -0.37 |
| SRB | T0 | 215 | 3.5 (2.6) | 0-10 | 107 | 3.9 (2.7) | | 0-10 | 108 | 3.2 (2.5) | 0-10 | | *z* = -1.83 |
| *Caregiver burden* | T2 | 176 | 3.6 (2.6) | 0-10 | 79 | 3.7 (2.6) | | 0-8.9 | 97 | 3.4 (2.5) | 0-10 | | *z* = -0.61 |
|  | T4 | 103 | 3.2 (2.4) | 0-8.8 | 47 | 3.4 (2.6) | | 0-8 | 56 | 3.0 (2.3) | 0-8.8 | | *z* = -0.60 |
| Guilt | T0 | 220 | 9.9 (4.2) | 5-22 | 109 | 9.7 (3.9) | | 5-21 | 111 | 10.2 (4.5) | 5-22 | | *z* = -0.75 |
| *Guilt in caregiving* | T2 | 179 | 9.8 (4.2) | 5-24 | 80 | 9.7 (4.4) | | 5-24 | 99 | 10.0 (3.9) | 5-20 | | *z* = -0.75 |
|  | T4 | 104 | 9.4 (4.2) | 5-20 | 48 | 9.2 (3.8) | | 5-20 | 56 | 9.6 (4.5) | 5-20 | | *z* = -0.03 |
| Conflict | T0 | 221 | 22.7 (7.4) | 10-44 | 109 | 22.0 (7.1) | | 10-41 | 112 | 23.4 (7.6) | 10-44 | | t(219)= 1.4 |
| *Conflict with staff* | T2 | 180 | 23.5 (7.9) | 10-43 | 81 | 22.8 (7.8) | | 10-43 | 99 | 24.1 (8.0) | 10-43 | | t(178)= 1.1 |
|  | T4 | 105 | 23.4 (7.3) | 10-41 | 48 | 22.3 (6.7) | | 10-41 | 57 | 24.2 (7.7) | 12-41 | | t(103)= 1.3 |

*Note*: **p* <0.05. ***p* <0.01, ****p* <0.005, *****p* <0.001; N=231 at baseline with n_intervention_=116 ; n_control_=115. Due to missing values and drop-out, the total per variable per time of assessment can differ. Comparisons were not controlled for multiple testing. ^†^ independent t-test, Mann Whitney U-test or Fisher’s exact test, ^‡^,distribution of the seasons of the observation periods was approximately the same in the intervention group and the control group, T0=baseline assessment, T1=1-month follow up, T2=3-month follow up, T3=6-month follow up, T4=12-month follow up, QUALID = Quality of Life in Late-Stage Dementia with lower scores representing better quality of life, GAIN = Gain in Alzheimer care INstrument, DS-DAT = Discomfort Scale-Dementia of Alzheimer Type, NPI-Q = Neuropsychiatric Inventory Questionnaire, ZBI = Zarit’s caregiver burden scale, SRB = Self-Rated Burden scale (single 0-10 scale), Guilt = Family Perceptions of Caregiving Role (FPCR) subscale ‘guilt’, Conflict = FPCR, subscale ‘conflict with staff'.
