# Supplementary material for: Namaste Care Family for people with dementia and family−A randomized controlled trial
Source: Alzheimers Dement. 2025 Jul 19;21(7):e70495. doi: 10.1002/alz.70495 (PMC12276077; doi:10.1002/alz.70495)
Supplement: Supplementary file 2 — Supporting Information [file ALZ-21-e70495-s003.docx]

**Supplementary materials**

Smaling HJA, Joling KJ, Rijnhart JJM, Twisk JWR, Achterberg WP, Francke AL, van der Steen JT. Namaste Care Family for people with dementia and family - a randomized controlled trial.

**Supplement II:** Background on cost-effectiveness

***A. Summary of the cost-effectiveness study by El Alili et al^1^.***

The cost-effectiveness study of the Dutch Namaste Care Family program^1^ was based on the current cluster-RCT that included 231 residents across 19 nursing homes. The primary outcomes of were residents’ Quality of Life in Late-Stage Dementia (QUALID) and the Gain in Alzheimer Care Instrument (GAIN) for family caregivers over 12 months of follow-up. Health states were measured using the three-level version of the EuroQol (EQ-5D-3L) which were translated into utilities. Quality-adjusted life years (QALYs) were calculated by multiplying the amount of time a participant spent in a specific health state with the utility score associated with that health state. Healthcare utilization costs were estimated using standard unit costs. Costs were measured from a societal perspective (secondary care costs, medication costs, family costs and Namaste Care Family costs).

The mean difference in QALYs between the groups was not significant. Total healthcare costs were lower in the Namaste-group compared to the control-group, but this difference was not significant (€ −1111, 95%CI: −4071 to 1246). Medication costs were the main contributor to this cost difference (€−1002, 95%CI: −3971 to 1268). The cost-utility analysis resulted in an incremental cost-effectiveness ratio (ICER) of – 315.67. This indicates that for each QALY that is gained, €315.67 is saved in the Namaste-group compared with the control-group. This suggested that the program could be considered cost-effective at commonly accepted willingness-to-pay thresholds.

***B. Reflection on economic implications of the effect of Namaste Care Family on pneumonia***

Treatment of pneumonia in nursing home residents with dementia varies across countries. Dutch nursing homes employ physicians who completed a three-year specialist training program to become a qualified so-called “elderly care” physician.^2^ They are responsible for the treatment of residents, and this model contributes to fewer (avoidable) hospitalizations compared to other countries.^3^ The physicians’ presence allows for close monitoring of diseases and health problems. Intercurrent diseases such as pneumonia, that may require hospitalization in other countries, are usually treated in the nursing home in the Netherlands.^2^ Prognosis is an important consideration in antibiotic treatment decisions for residents with dementia and pneumonia; Dutch physicians prefer not to treat with antibiotics when this is probably futile.^4^ The physicians are also inclined to withhold curative treatment in residents with severe dementia and pneumonia, and more often when the prognosis is poor.^5^ For example, care for nursing home residents with lower respiratory tract infection and dementia in the United States of America was more intensive than care for Dutch residents, particularly in residents with severe dementia.^6^

Hospitalization due to pneumonia is common elsewhere in Europe and contributes significantly to healthcare costs.^7^ It is possible that the lower, though not statistically significant, healthcare costs observed in the cost-effectiveness study by El Alili et al.^1^ may be partially attributed to the program’s impact on pneumonia. Notably, no cases of pneumonia were reported in the Namaste group during the final six months of the study. Future research should examine the impact of Namaste Care on pneumonia, including the costs related to pneumonia, in other countries. Although no statistically significant difference in healthcare costs was found between the two groups in the Dutch setting, this ﬁnding may be culturally bound. The Dutch long-term care system is distinctive in its use of specialized physicians working in nursing homes who guide clinical decision making.^3^ Physicians in other countries may have different perspectives on medical futility and may be influenced by other factors when making treatment decisions.^8^

**References**

1. El Alili M, Smaling HJA, Joling KJ, Achterberg WP, Francke AL, Bosmans JE, van der Steen JT. Cost-effectiveness of the Namaste care family program for nursing home residents with advanced dementia in comparison with usual care: a cluster-randomized controlled trial. BMC Health Serv. Res. 2020 Sep 4;20(1):831. doi: 10.1186/s12913-020-05570-2.
2. Koopmans RT, Lavrijsen JC, Hoek JF, Went PB, Schols JM. Dutch elderly care physician: a new generation of nursing home physician specialists. J Am Geriatr Soc. 2010 Sep;58(9):1807-9. doi: 10.1111/j.1532-5415.2010.03043.x
3. Helton MR, van der Steen JT, Daaleman TP, Gamble GR, Ribbe MW. A cross-cultural study of physician treatment decisions for demented nursing home patients who develop pneumonia. Ann. Fam. Med. 2006 May 1;4(3):221-7.
4. van der Steen JT, Helton MR, Ribbe MW. Prognosis is important in decisionmaking in Dutch nursing home patients with dementia and pneumonia. Int J Geriatr Psychiatry. 2009 Sep;24(9):933-6. doi: 10.1002/gps.2198.
5. van der Steen JT, Kruse RL, van der Wal G, Mehr DR, Ribbe MW. Behandeling van pneumonie bij verpleeghuispatiënten met ernstige dementie: terughoudender beleid in Nederland en actiever beleid in de verenigde Staten naarmate de prognose ongunstiger is [Treatment of pneumonia in nursing home residents with severe dementia: for residents with poor prognosis, a more reserved approach in The Netherlands and more active treatment in the United States]. Ned Tijdschr Geneeskd. 2007; 21;151(16):915-9.
6. van der Steen JT, Kruse RL, Ooms ME, Ribbe MW, van der Wal G, Heintz LL, Mehr DR. Treatment of nursing home residents with dementia and lower respiratory tract infection in the United States and The Netherlands: an ocean apart. J Am Geriatr Soc. 2004 May;52(5):691-9. doi: 10.1111/j.1532-5415.2004.52204.x.
7. Tsoumani E, Carter JA, Salomonsson S, Stephens JM, Bencina G. Clinical, economic, and humanistic burden of community acquired pneumonia in Europe: a systematic literature review. Expert Rev. Vaccines. 2023;22(1):876-84.
8. van der Maaden T, Hendriks SA, de Vet HC, Zomerhuis MT, Smalbrugge M, Jansma EP, Koopmans RT, Hertogh CM, van der Steen JT. Antibiotic use and associated factors in patients with dementia: a systematic review. Drugs Aging. 2015;32(1):43-56. doi: 10.1007/s40266-014-0223-z.
